# Case Report: Electroencephalography-informed low-frequency repetitive transcranial magnetic stimulation targeting the supplementary motor area in severe functional movement disorder

**DOI:** 10.3389/fpsyt.2026.1959751

**Published:** 2026-10-16

**Authors:** Chiaki Takahashi, Takashi Asahi, Motoki Kanda, Yoshihiko Ohta, Isao Matsushita

**Affiliations:** 1 Department of Physical and Rehabilitation Medicine, Kanazawa Medical University, Uchinada, Japan; 2 Department of Neurosurgery, Kanazawa Neurosurgical Hospital, Nonoichi-shi, Japan; 3 Department of Rehabilitation, Kanazawa Medical University Hospital, Uchinada, Japan; 4 Central Clinical Laboratory, Kanazawa Medical University Hospital, Uchinada, Japan

**Keywords:** electroencephalography, functional connectivity, functional movement disorder, rehabilitation, repetitive transcranial magnetic stimulation, transcranial direct current stimulation

## Abstract

**Background:**

Functional movement disorder (FMD) is increasingly being recognized as a condition associated with abnormal brain network connectivity; however, effective treatment strategies remain limited.

**Methods:**

We report the case of a patient with severe FMD that developed following an occupational injury and was characterized by functional tremors accompanied by migraines and prominent autonomic symptoms. Based on electroencephalography (EEG) network analysis, low-frequency repetitive transcranial magnetic stimulation targeting the supplementary motor area was administered, followed by staged rehabilitation incorporating visual feedback.

**Results:**

The combined intervention reduced tremors and improved gait speed, activities of daily living, and EEG network connectivity.

**Conclusion:**

This case highlights the potential utility of combining EEG-guided neuromodulation with staged rehabilitation for the treatment of FMD and supports further investigation of this approach.

## Introduction

Functional movement disorder (FMD) is a subtype of functional neurological disorder (FND) with an annual incidence of 4–5 per 100,000 people and a prevalence of approximately 50 per 100,000 individuals (1). FMD is frequently encountered in routine neurology clinics and pediatric departments. It encompasses a wide range of movement disorders, including functional tremors, myoclonus, dystonia, gait disturbances, and parkinsonism, and functional tremors represent the most common clinical presentation (2). Historically, FMD has been considered a psychogenic disorder (3). Patients with FMD often report psychological stressors or physical trauma such as motor vehicle accidents, as potential precipitating factors (2). Approximately 70% of the affected individuals are female, with a mean age at onset of approximately 40 years, but the proportion of male patients increases with age (4).

Advances in multimodal neuroimaging over the past few decades have suggested that FMD is characterized by aberrant functional connectivity within brain networks (5). In patients with functional tremors, the increased activity within the motor–limbic network involving the anterior cingulate cortex (ACC) and paracingulate cortex and reduced activation of the right temporoparietal junction (rTPJ) that characterize the subtype in the present case have been reported. Reduced rTPJ activation is believed to contribute to an impaired sense of agency, leading patients to experience movements outside their voluntary control (6). In addition, increased resting-state regional cerebral blood flow has been reported in the left inferior frontal gyrus and insular cortex (7).

Beyond motor symptoms, FMD has been associated with alterations in autonomic and endocrine functions including reduced vagal tone (8, 9). Recent studies have reported diminished vagal tone in these patients, suggesting altered autonomic regulation and stress resilience (10). However, the profound autonomic instability in patients with FMD remains poorly characterized.

Here, we describe the clinical course of a patient with severe FMD accompanied by prominent autonomic symptoms, including hypertension, recurrent fever, and migraine, who underwent electroencephalography (EEG)-informed low-frequency repetitive transcranial magnetic stimulation (rTMS) targeting the supplementary motor area (SMA), followed by staged rehabilitation.

## Case presentation

A 39-year-old man with an unremarkable medical history presented to our hospital. His family history was unremarkable. At the age of 36, a load weighing more than 100 kg fell toward him while he was at work. While attempting to catch the object, he was dragged and his body was forcibly twisted to the left. The patient immediately developed severe neck and lower back pain, and was unable to move. He was transported to a local emergency department, where computed tomography (CT) of the chest, abdomen, thoracic spine, and lumbar spine revealed no significant abnormalities, and he was subsequently discharged.

The following day, worsening of the posterior neck and lower back pain prompted a consultation with the orthopedic department of another hospital. Lumbar magnetic resonance imaging (MRI) revealed right sacral bone contusion and lumbar disc herniation. Conservative treatment, consisting primarily of pain management and rehabilitation, was initiated. A sacral nerve block was also performed; however, its therapeutic effect was limited.

One month post-injury, the patient developed an unexplained fever (37–38 °C), abnormal sensations and tremors involving the trunk and all four limbs, sensation of head heaviness, and severe worsening pain in the lower back. Consequently, he was referred to a tertiary neurology department two months post-injury. Neurological findings revealed neither muscle weakness nor abnormalities in the deep tendon reflexes. Because of the unexplained hypertension and persistent fever, autoimmune diseases and pheochromocytoma were considered in the differential diagnosis. However, comprehensive laboratory investigations including serum catecholamine measurements revealed no significant abnormalities. Neurophysiological examinations, including nerve conduction studies, repetitive nerve stimulation, and somatosensory-evoked potentials of both the upper and lower extremities, revealed no abnormalities. Autonomic function was assessed using the head-up tilt test and sympathetic skin response, neither of which revealed any abnormalities; however, heart rate variability was not assessed. Brain MRI revealed no structural abnormalities. Cervical MRI demonstrated mild C6/7 foraminal stenosis associated with a small C7 disc herniation without spinal cord compression, which was considered insufficient to explain the generalized movement disorder of the patient. Since the symptoms developed after the traumatic injury and were accompanied by autonomic manifestations, Barré–Lieou syndrome was considered the most likely diagnosis. Due to persistent hypertension, further cardiological evaluation with cardiac CT and echocardiography revealed dilated cardiomyopathy. Sacubitril/valsartan (200 mg/day), empagliflozin (10 mg/day), and vericiguat (2.5 mg/day) were administered to treat the heart failure associated with dilated cardiomyopathy.

During follow-up at a local hospital, the patient developed a migraine that was not present before the injury. Therefore, metoprolol (120 mg/day) and lomerizine (10 mg/day) were administered, with sumatriptan (50 mg) used as needed for acute migraine attacks. All regularly scheduled medications were maintained at unchanged doses throughout the rTMS treatment and rehabilitation periods. The patient also underwent prolonged rehabilitation without any significant improvement in the motor symptoms. Subsequently, a movement disorder specialist established the diagnosis of FMD based on positive clinical signs, including marked variability, distractibility, entrainment during rhythmic tapping, co-contraction, and incongruence with known organic movement disorders. The patient underwent cognitive behavioral therapy; however, no clinically meaningful improvement in motor symptoms was observed. He was ultimately referred to our hospital for evaluation of eligibility for rTMS treatment.

At presentation to our hospital, generalized co-contraction was absent while the patient was seated, but became pronounced during voluntary limb movements accompanied by tremors. Even simple elevation of both upper limbs required considerable effort and resulted in shortness of breath. While walking, upper limb tremors were absent when the patient used a cane. However, knee tremor, intermittent knee buckling, and excessive knee extension were observed. The tremors showed marked variability depending on the posture and type of upper limb movement performed, which is consistent with the characteristic features of FMD. Video 1 shows the pre-treatment recording on the left and the post-treatment recording on the right.

EEG was performed on admission and repeated after the intervention. Resting-state EEG was recorded using a Neurofax EEG-1260 (Nihon Kohden Corp., Tokyo, Japan), with electrodes placed according to the international 10–20 system. The averaged bilateral auricular electrodes (A1 and A2) served as references, and the ground (Z) electrode was placed at the nasion. Recordings were acquired under eyes-closed, resting conditions using a monopolar montage (sampling rate: 200 Hz, band-pass filter: 1–45 Hz). They lasted for approximately 6 min. After visual inspection of the continuous recording, the initial and terminal portions containing recording-related or movement artifacts were excluded, and a continuous 5-min segment with minimal artifacts was selected for subsequent analysis. Power spectral density (PSD) and weighted phase lag index (wPLI) analyses (11) were performed using MNE-Python (version 1.12.1) and MNE-connectivity (version 0.7.0) (12, 13). PSD was estimated using Welch’s method with 2-s segments and 50% overlap between adjacent segments. The fast Fourier transform length was set equal to the segment length, yielding a frequency resolution of 0.5 Hz. The PSD estimates obtained from individual segments were averaged arithmetically. The resulting spectra were linearly interpolated onto a frequency grid ranging from 1.5625 to 46 Hz. For each electrode, the PSD at each frequency was normalized by the sum of the PSD values across the analyzed frequency range to obtain the normalized PSD such that the sum of the normalized PSD values within each electrode was equal to 1.

For functional connectivity analysis, the EEG data were notch-filtered at 50 Hz and band-pass filtered between 1 and 40 Hz using zero-phase finite impulse response filters, followed by reference to the common average. Independent component analysis was performed using the Fast algorithm with 15 components. The components corresponding to ocular artifacts were identified using Fp1 as a surrogate electrooculography channel and removed. Eighteen electrodes (Fz, Fp1, F3, F7, T3, C3, T5, P3, O1, O2, P4, T6, C4, T4, F8, F4, Fp2, and Cz) were selected, and the cleaned continuous EEG was divided into non-overlapping 4-s epochs. Functional connectivity was quantified using the debiased estimator of the squared weighted phase lag index (wPLI2-debiased) with a Fourier-based spectral estimation. Connectivity was calculated for all electrode pairs and averaged within the theta (4–8 Hz), alpha (8–13 Hz), and beta (13–30 Hz) frequency bands. No connectivity threshold was applied to the quantitative analysis. Given the single-case design, pre- and post-intervention EEG changes were evaluated descriptively, and inferential statistical testing was not performed. Accordingly, corrections for multiple comparisons were not applied.

Normalized PSD analysis demonstrated increased theta-band power at Cz (**Figure 1**). In addition, wPLI analysis revealed excessive functional connectivity centered on C4 in the alpha and beta frequency bands (**Figure 2**). Previous functional neuroimaging studies have demonstrated reduced functional connectivity between the rTPJ, a key region involved in the sense of agency, and sensorimotor regions, including the bilateral SMA, in patients with FMD (14). Given the bilateral and widespread nature of the patient’s motor symptoms, including bilateral tremor and co-contraction, we considered the SMA as a clinically relevant target that allowed simultaneous modulation of the bilateral medial motor regions. The scalp EEG findings, including relatively prominent theta-band activity at Cz and altered C4-centered functional connectivity, were considered supportive of motor network dysfunction but were not interpreted as directly localizing the abnormality to the SMA. Based on these clinical, neurophysiological, and previously reported network findings, the bilateral SMA was selected as the stimulation target.

**Figure 1:**
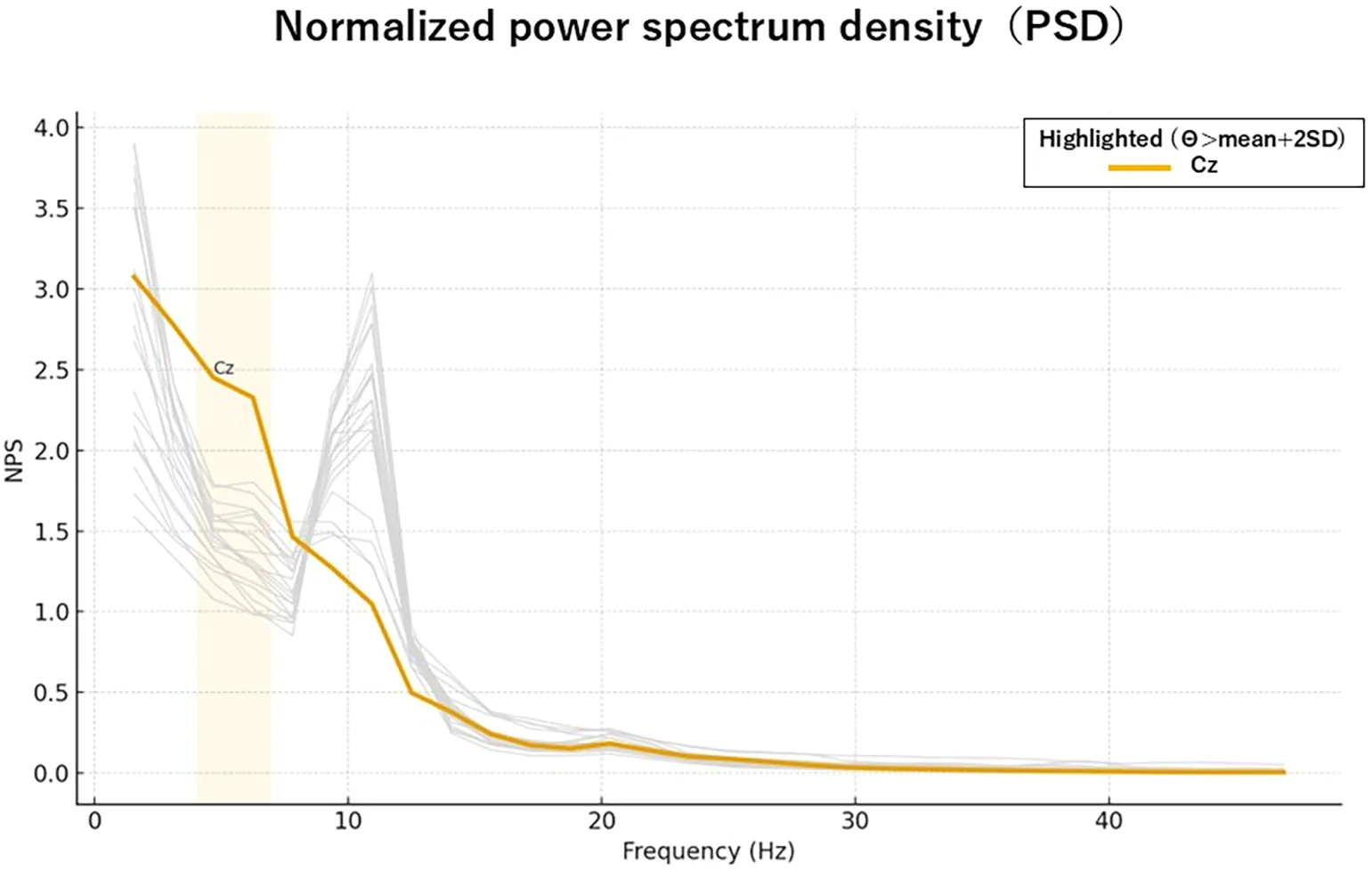
The normalized power spectrum density (PSD) was derived from EEG data recorded during the initial evaluation of the patient at our hospital. The x-axis represents the frequency (Hz), and the y-axis represents the NPS. Gray lines indicate the normalized PSD of the individual EEG electrodes, whereas the orange line represents the Cz electrode. The shaded area indicates the θ frequency band (4–7 Hz). The Cz electrode demonstrated θ-band power exceeding the within-subject mean ± 2 of all electrodes and was therefore highlighted.

**Figure 2:**
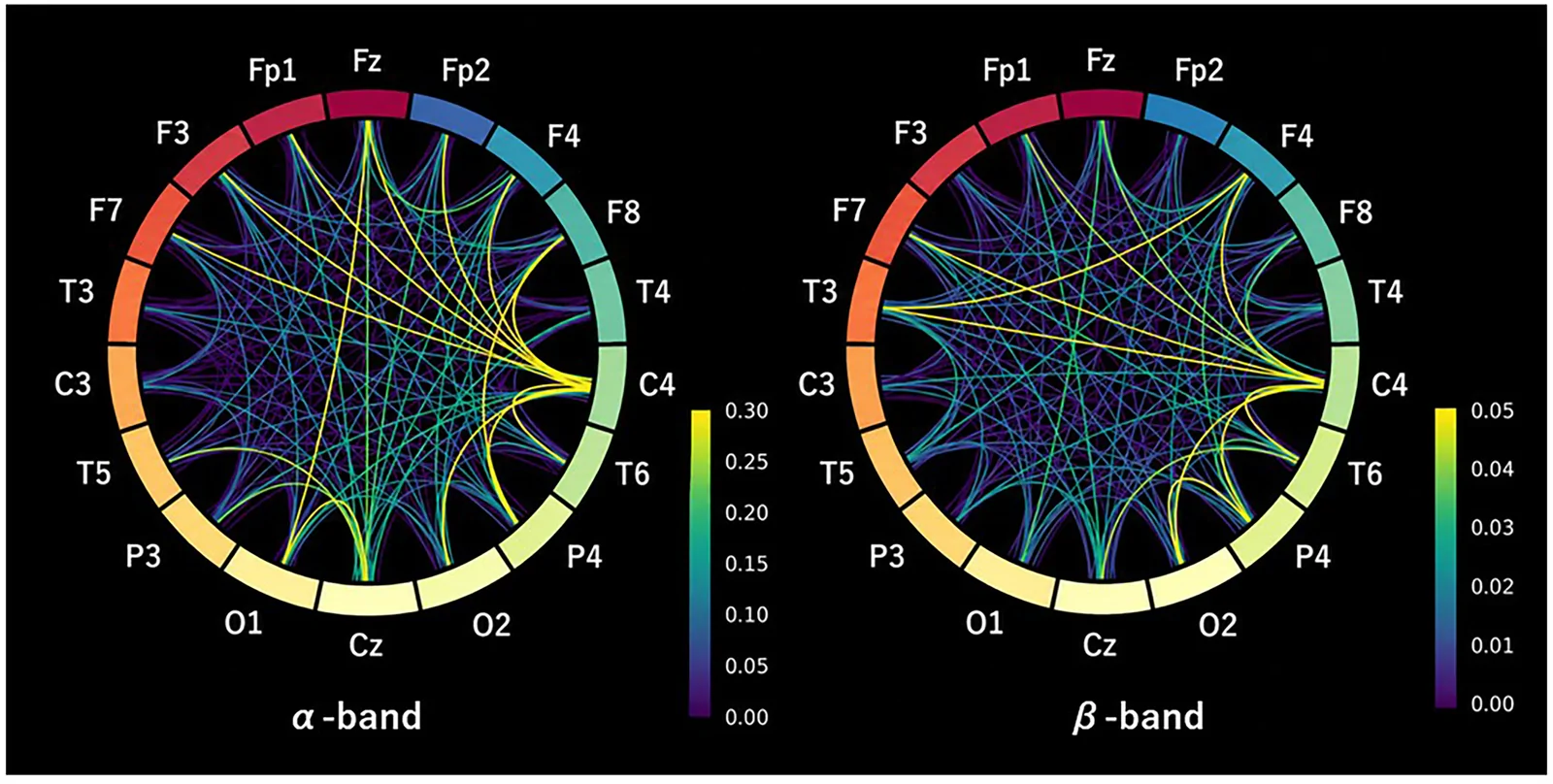
Connectivity circle generated from weighted phase lag index (wPLI) analysis of EEG recordings obtained during the initial evaluation of the patient at our hospital. Functional connectivity in the α (8–13 Hz) and β (13–30 Hz) frequency bands. Pre-treatment network demonstrates a C4-centered hub-like organization, with excessive functional connectivity concentrated around C4.

Based on this rationale, transcranial direct current stimulation (tDCS) was initially administered using a Compact DC Stimulator GD-800 (OG Wellness Technologies Co., Ltd., Okayama, Japan). Assuming hyperexcitability at Cz, a 5 × 7 cm rectangular cathodal electrode was placed over Cz to suppress cortical excitability, and stimulation was delivered at 2 mA for 20 min; however, no clinical improvement was observed. Subsequently, bilateral cathodal stimulation of the primary motor cortex (M1) was performed simultaneously using the two stimulators. However, this approach was ineffective.

We subsequently initiated rTMS targeting the SMA using a MagPro R30 stimulator (MagVenture A/S, Farum, Denmark) based on this rationale. The target was defined as a point 2.5 cm anterior to Cz, corresponding to the SMA. Low-frequency rTMS was delivered at 1 Hz and 90% of the resting motor threshold, with 900 pulses administered over 15 min per session. Treatment was performed every 2–3 days during the 2-week hospitalization for six sessions (5,400 pulses in total). Immediately after the first rTMS session, tremors associated with bilateral upper- and lower-limb elevations were noticeably reduced on visual clinical observation. The symptoms were quantitatively assessed using the Simplified Functional Movement Disorders Rating Scale (S-FMDRS) (15). The severity score decreased from 14/27 before stimulation to 11/27 at discharge, whereas the duration score remained unchanged at 15/27. A reduction in tremors following rTMS was evident, but the patient showed little subjective awareness of the improvement, consistent with previous reports. Therefore, visual feedback was provided by reviewing videos recorded before and after treatment to facilitate the recognition of clinical improvement. The patient received 60 min of daily physical and occupational therapy during hospitalization. During the early phase of hospitalization, excessive tremors occurred immediately after the initiation of voluntary movements, making active exercise therapy difficult. Therefore, during the first week, rehabilitation focused primarily on relaxation techniques and management of low back pain. From the second week onward, visual feedback using mirror-based and simple task-oriented movement exercises were gradually introduced as tolerated. At the final assessment, tremors in both the upper and lower limbs did not completely resolve, but both the 10-m walking time (11.22 s to 8.59 s) and Timed Up and Go test time (clockwise; 13.87 s to 9.47 s) showed improvement compared with the baseline (Video 1-Right). EEG performed immediately before discharge demonstrated modulation of alpha- and beta-band functional connectivity patterns with reduced relative prominence of the C4-centered connectivity (**Figure 3**). In the alpha band, the mean C4 connectivity increased modestly from 0.127 to 0.150, whereas the global mean connectivity increased from 0.048 to 0.102. Among the 17 electrode pairs involving C4, the wPLI increased for 12 pairs and decreased in 5 pairs. Consequently, despite the increase in mean C4 connectivity, the ratio of C4 mean connectivity to global mean connectivity decreased from 2.67 to 1.46, and the C4 node-strength rank changed from first to third among the 18 electrodes. In the beta band, the mean C4 connectivity decreased from 0.0349 to 0.0263 and the C4 node strength decreased from 0.593 to 0.448. Among the 17 electrode pairs involving C4, wPLI increased in 6 pairs and decreased in 11 pairs. The C4/global connectivity ratio decreased from 2.95 to 1.29, and the node strength rank changed from first to fifth. These pairwise changes were evaluated descriptively and not subjected to inferential statistical testing. Summary metrics of C4-centered connectivity are presented in **Supplementary Table S1**, and detailed pairwise wPLI values are provided in **Supplementary Table S2**. At the 1-month follow-up, clinical improvements were sustained, and the patient reported improvements in activities of daily living, lower back pain, and joint pain, indicating a high level of satisfaction with the treatment. However, the tremor recurred two months later.

**Figure 3:**
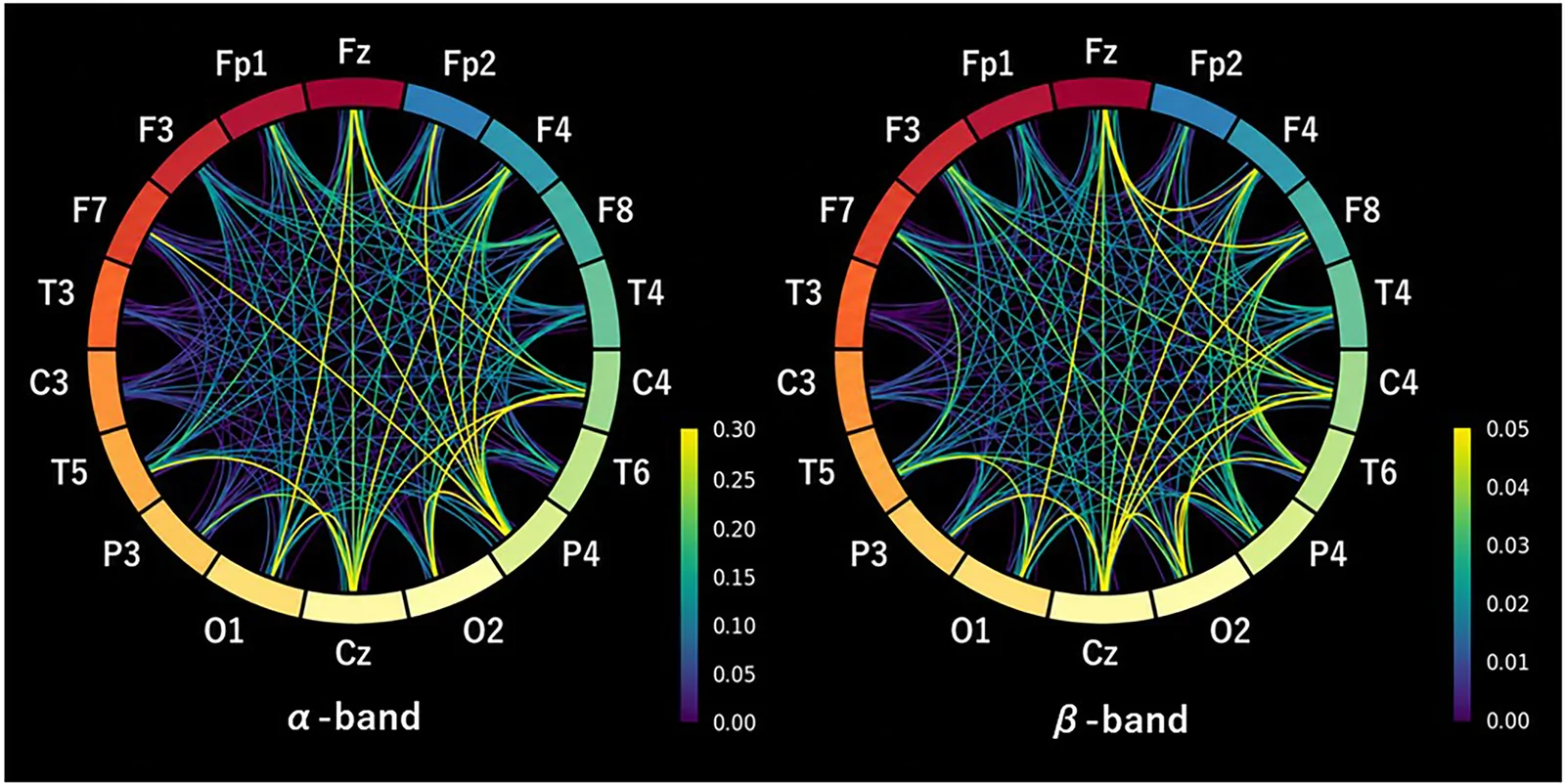
Connectivity circle generated from wPLI analysis of EEG recordings obtained during the patient evaluation after low-frequency rTMS. Functional connectivity in the α- and β-frequency bands is shown. Compared with the pre-treatment findings, the excessive C4-centered hub-like organization was attenuated, and functional connectivity became more widely distributed across the network.

## Discussion

This case report describes a patient with severe post-traumatic FMD characterized by marked generalized co-contraction and tremors during voluntary movement, accompanied by autonomic and systemic symptoms. Barré–Lieou syndrome is currently regarded as a historical and controversial diagnostic concept (16); however, it was initially considered because of the patient’s post-traumatic neck pain and prominent autonomic symptoms. Furthermore, EEG analysis demonstrated relatively prominent theta band power at Cz, together with a C4-centered pattern of functional connectivity in the alpha and beta bands. The changes in theta band activity and functional connectivity observed after low-frequency rTMS targeting the SMA are particularly noteworthy.

Traditionally, FMD has been considered a psychogenic disorder. However, accumulating evidence suggests that it is better understood as a disorder of the motor control networks involving abnormalities in motor preparation, attentional processing, sense of agency, and salience networks (5, 7, 17, 18). Increased activity in the motor/emotional processing network involving the ACC and paracingulate cortex, together with reduced rTPJ activation, has been reported in patients with FMD (6). Maurer et al. reported reduced resting-state functional connectivity between the rTPJ and SMA, sensorimotor cortex, cerebellum, and insular cortex in patients with FMD, suggesting that an impaired sense of agency may contribute to the pathophysiology of this disorder (10). In addition, one study reported reduced local activity within the SMA in patients with FMD (19), but another demonstrated increased functional connectivity between the primary motor cortex and higher-order motor networks, including the SMA, TPJ, and prefrontal cortex (20). In the present case, we selected the SMA as the stimulation target for rTMS. The relatively prominent functional synchronization centered in the right C4 region with the frontal and parietal cortices may reflect altered integration within the motor control network. The reduced relative prominence of the C4-centered connectivity following rTMS suggests that SMA stimulation may modulate functional connectivity beyond the stimulation site. Despite the apparent visual reduction in tremors after rTMS, the patient initially showed little subjective awareness of improvement. However, he was able to visually recognize the improvement and became increasingly aware of the ease of performing voluntary movements after reviewing the video recordings obtained before and after treatment. For FMD, the motor execution and processes involved in recognizing and self-evaluating movements are thought to be impaired. The clinical course observed in the present case suggests that external visual feedback may have helped recalibrate the patient’s perception of his movements, thereby improving the accuracy of self-monitoring. Consistent with this hypothesis, previous studies have shown that neurofeedback training targeting the agency network, including the rTPJ, can enhance the explicit sense of agency (i.e., the subjective feeling of being the agent of one’s actions) in patients with FND (21). Similarly, the video-based feedback used in the present case may have facilitated reconstruction of the sense of agency, thereby contributing to the observed clinical improvement.

In addition to the motor manifestations, the patient had diverse autonomic symptoms, including hypertension, unexplained fever, and profound fatigue. Dilated cardiomyopathy was independently diagnosed by the Cardiology Department, and the patient required treatment for heart failure. Recent studies have increasingly recognized alterations in autonomic function in patients with FMD in addition to abnormalities in motor control networks. Reduced vagal tone, which reflects impaired parasympathetic function, has been proposed as a potential mechanism underlying autonomic dysregulation and diminished stress resilience in patients with FMD (9, 10). Several studies have reported reduced heart rate variability in patients with FMD, as evidenced by a lower root mean square of successive differences, an electrocardiographic marker of cardiac vagal tone (10). Furthermore, a tendency toward increased heart rate has been reported in pediatric patients with FMD (9). In the present case, the head-up tilt test and sympathetic skin response revealed no abnormalities, and heart rate variability was not assessed. While motor and autonomic symptoms developed during the same clinical course, objective autonomic dysfunction was not demonstrated, and a causal relationship between FMD-related autonomic alterations and independently diagnosed cardiomyopathy could not be established.

The patient had no history of migraine before the onset of FMD but subsequently developed new-onset migraine. Migraine is a common comorbidity of FMD. One study reported that 83% of patients experienced chronic headache, of whom 58% met the diagnostic criteria for migraine (9). Migraines generally precede the onset of FMD, but only a few cases in which FMD precedes the development of migraines have been reported (22). Recent studies have demonstrated abnormalities in salience, limbic, and autonomic regulatory networks in patients with FMD. Similar network dysfunctions and altered sensory processing have also been implicated in the pathophysiology of migraines (23). The temporal associations of the onset of FMD with autonomic/systemic symptoms and migraines in the present case is noteworthy and may suggest partially overlapping network mechanisms involving sensory processing and autonomic regulation.

Pre-treatment EEG analysis demonstrated a relatively prominent theta band power at Cz. Theta band activity is implicated in attentional control, executive function, and motor preparation and interacts with alpha-band oscillations to regulate cognitive and emotional processes (24–26). Furthermore, the wPLI analysis revealed a C4-centered pattern of functional connectivity in the alpha and beta frequency bands. In the alpha band, widespread connectivity between C4 and the frontal and parietal cortices may reflect altered synchronization within distributed motor-related networks. In contrast, the beta-band connectivity may reflect altered synchronization within motor-related networks (27). However, its specific functional significance cannot be determined from the present wPLI analysis alone. Bereitschaftspotential and event-related desynchronization are widely used as neurophysiological markers of motor preparation, and findings from these measures suggest that abnormalities in motor preparatory processes contribute to the pathophysiology of FMD (28). These measures were not assessed in the present case and are discussed here solely in the context of previous literature.

Cathodal tDCS over Cz and bilateral M1 stimulation failed to produce any clinical improvement. In contrast, low-frequency rTMS targeting the SMA resulted in an immediate reduction in tremors after the first session. Residual tremors persisted at the final assessment, but clinically meaningful improvements were observed in clinical and functional measures, including the 10-m walking test, Timed Up and Go test, S-FMDRS, and activities of daily living. Since prolonged rehabilitation before referral failed to improve the symptoms of the patient, the immediate response to the first rTMS session suggested that it may have facilitated subsequent motor relearning during rehabilitation. Moreover, post-treatment EEG demonstrated reduced relative prominence of C4-centered functional connectivity, indicating a reconfiguration of the functional connectivity pattern. These findings raise the possibility that rTMS may not simply suppress symptoms but rather modulate motor network organization, thereby facilitating subsequent motor relearning during staged rehabilitation. This interpretation is consistent with the concept of motor priming in which noninvasive brain stimulation may enhance neuroplasticity and optimize the effects of subsequent rehabilitation (29).

In addition, rehabilitation initially prioritized relaxation techniques and pain management as severe tremors prevented active exercise therapy. Following the initial tremor reduction, mirror-based visual feedback and simple task-oriented motor training were introduced in a stepwise manner. The clinical course in this case raises the hypothesis that neuromodulation followed by staged motor relearning warrants further investigation as a therapeutic approach for treating severe FMD. Nevertheless, the specificity and generalizability of these electrophysiological findings, including alterations in functional connectivity, remain uncertain because this is a single-case report. Future studies with larger cohorts are warranted to clarify the relationship between these network alterations and the mechanisms underlying neuromodulation in patients with FMD.

## Conclusion

In this patient with FMD presenting with functional tremors, altered motor network connectivity and autonomic symptoms were accompanied by clinical and electrophysiological changes following low-frequency rTMS targeting the SMA combined with a staged rehabilitation program. These findings are consistent with the concept of FMD as a motor network dysfunction and suggest that an individualized therapeutic strategy combining EEG-informed neuromodulation with staged rehabilitation warrants further investigation for selected patients with FMD.

## Data Availability

The raw data supporting the conclusions of this article will be made available by the authors, without undue reservation.
